# Cytokine levels contribute to the pathogenesis of minimal hepatic encephalopathy in patients with hepatocellular carcinoma via STAT3 activation

**DOI:** 10.1038/srep18528

**Published:** 2016-01-13

**Authors:** Hao Wu, Ning Li, Ronghua Jin, Qinghua Meng, Peng Chen, Guoxian Zhao, Rui Wang, Li Li, Wei Li

**Affiliations:** 1 Center for Infectious Diseases, Beijing You’an Hospital, Capital Medical University, Beijing 100069, China; 2 Center for Liver Transplantation, Beijing You’an Hospital, Capital Medical University, Beijing 100069, China; 3 Department of Severe Liver Disorders, Beijing You’an Hospital, Capital Medical University, Beijing 100069, China; 4 Minimally Invasive Therapy Center of Liver Cancer, Beijing You’an Hospital, Capital Medical University, Beijing 100069, China

## Abstract

Hepatocellular carcinoma (HCC) patients were grouped according to the degree of encephalopathy, with healthy volunteers as controls. We investigated clinical presentation, protein and mRNA expression of 14 cytokines, and activation of six STAT proteins, the downstream signaling mediators. Levels of all 14 cytokines were significantly elevated in HCC patients with clinical hepatic encephalopathy. Statistical analysis showed that levels of IL-1β, IL-6, IFNγ, IL-17α, IFNλ2 and IFNλ3 were correlated with minimal hepatic encephalopathy (MHE). Multivariate regression analysis identified serum IL-6, IFNλ3 and IL-17α as independent risk factors for MHE. Increased mRNA levels of IL-6 and IFNγ were associated with MHE. Among the STAT proteins examined, only STAT3 was elevated in MHE. Treatment with a STAT3 inhibitor protected neurons from cytokine-induced apoptosis *in vitro*. In conclusion, this study identified potential biomarkers for MHE in HCC. The cytokines investigated may induce neural apoptosis via STAT3 in the pathogenesis of MHE in HCC.

Hepatocellular carcinoma (HCC) is one of the most common cancers in the world, and a major cause of death in areas with a high incidence of viral hepatitis^1^. Hepatic encephalopathy (HE) is a neuropsychiatric syndrome commonly seen at the end stage of HCC. HCC patients with HE have a high mortality rate^2^,^3^. Minimal HE (MHE) is the early and mild stage of HE^4^,^5^, and cannot be detected by general clinical examination, but can be revealed using specific neuropsychological examinations^6^,^7^. The psychometric HE score (PHES), which is the sum score from the five sub-tests of the PSE (Portosystemic Encephalopathy)-Syndrome-Test, is generally considered the “gold standard” in the diagnosis of MHE^6^,^8^. However, it is a time-consuming test, and requires adjustment for age and education level. A more convenient and accurate approach is needed.

Serum cytokines are key mediators for many physiological and pathological modulations including inflammation, cancer development, human nervous system development, bidirectional signal transduction between the central and peripheral nervous systems, and cognitive processes, amongst others^9^,^10^. Under most physiological conditions, most cytokine levels are low, but they can be elevated up to hundreds of times their basal levels in pathological conditions. Therefore, cytokines are good markers for the onset and progression of many illnesses. Regulation of expression and secretion of cytokines and their receptors have been described in various studies involving patients with severe liver conditions or HE^11^,^12^,^13^,^14^,^15^. For instance, circulating blood IL-1β, IL-1Ra, IL-2, IL-4, IL-6, IL-8, IL-10, IL-12, IFNγ, IL-17a and IL-18 concentrations were elevated in patients with liver cancers and HE^11^,^12^,^13^,^16^,^17^,^18^,^19^. Hyperammonemia was also suggested to contribute to the development of HE, and a correlation to levels of IL-6 in MHE has been reported^20^. This evidence suggests potential key roles of certain circulating cytokines in diagnosis and management of HE. However, the definite correlations between cytokines and MHE still need to be investigated, especially in HCC patients.

The objective of this study was to analyze the clinical and laboratory data of a group of patients with HCC, and to evaluate the correlations between serum cytokine levels and MHE. This aim of this study was to identify serum markers and potential pathogenetic mechanisms of MHE in patients with HCC in order to facilitate more accurate diagnosis of MHE, and facilitate early intervention that could potentially reduce mortality.

## Materials and Methods

### Study population

Our study population consisted of 121 HCC patients who were treated at our institution from April 2010 to July 2014. Sixty healthy controls were included in this study. The exclusion criteria included history of infectious diseases, autoimmune diseases, allergic response, immune deficiency disorder, diabetes, psychiatric illness, malignancy, severe cognitive impairment, or a systemic or CNS infection two weeks before sample collection. The diagnosis of HCC was determined by liver biopsy with imaging tests such as computed tomography (CT) or magnetic resonance imaging (MRI).

Subjects involved in this study were grouped as follows: (1) Controls: healthy volunteers without liver disorders; (2) CHE (clinical hepatic encephalopathy): HCC patients with a clinical hepatic encephalopathy diagnosis based on the West Haven criteria; (3) HCC patients without CHE were divided into two subgroups: (a) MHE (minimal hepatic encephalopathy) and (b) No MHE patients: patients were diagnosed by performing the psychometric test (PHES battery, see below for further details).

None of the involved patients had undergone a transjugular intrahepatic portosystemic shunt, nor did they have any history of spontaneous bacterial peritonitis. All patients were on normal diet, except for those with ascites who were on hyposodic diet, and for those with CHE who were on protein-restricted diet.

Psychometric tests were performed immediately following a standard history and physical examination. Within the same day, blood was drawn for routine laboratory investigations and cytokine level determination.

The Beijing You’an Hospital Ethics Committee approved the study, and all involved patients gave written informed consent for their clinical data and samples (blood, serum) to be used in this study. All experiments in this study were performed in accordance with the human experimentation guidelines of the People’s Republic of China.

### Cell Culture

Human neuronal cells were purchased from Procell Inc. (Wuhan, Hunan Province, China). The cells were maintained and grown as described in a previous publication^21^.

### Determination of Cytokine levels

Blood samples were collected by venipuncture into EDTA-anticoagulation vials. Plasma was harvested from the collected samples within 30 min and stored at −80 °C for subsequent cytokine analysis. Concentrations of IL-2, IL-4, IL-6, IL-8, IL-10, IFNγ, GM-CSF, TNFα (all antibodies from Bio-Rad, USA), IL-17a (PeproTech, Rocky Hill, NJ, USA), IL-1β (Bender MedSystems, Vienna, Austria), IL1Ra (Cytoscreen, Biosource, Belgium), IFNλ1, IFNλ2, IFNλ3, IFNλ4 (eBioscience, CA, USA), and IL-23 (Invitrogen, Carlsbad, CA, USA) were measured by ELISA according to manufacturers’ instruction^16^,^22^,^23^. ELISAs were performed in duplicate.

### TUNEL assay

Neuronal cells were stimulated with IL-1β, IL-6 (eBioscience, CA, USA), IFNλ3 (Novus, CO, USA), IFNγ (Sino Biological, Beijing, China) and IL-17a (eBioscience, CA, USA) for 72 hrs. TUNEL assays to determine DNA fragmentation in apoptotic cells were performed according to the manufacturer’s protocol (Promega). In brief, 3−5 × 10^6^ cells were briefly trypsinized, washed twice with cold PBS, fixed in 4% paraformaldehyde at 4 °C for 20 min, and washed again with PBS before permeabilization with 0.5 ml 0.5% saponin at 22 °C for 5 min. The cells were washed with PBS, incubated with 80 μl equilibration buffer at 22 °C for 5 min, washed with PBS again, then resuspended in 50 μl Nucleotide Mix and incubated in the dark at 37 °C for 1 h. Cells were washed again with PBS and analyzed by fluorescence microscopy.

### Immunofluorescence staining and flow cytometry analysis

Neuronal cells were first fixed and permeabilized using BD Cytofix/Cytoperm Fixation and Permeability Solution (BD Pharmingen, San Jose, CA), followed by staining for intracellular proteins, and fixation with 2% formaldehyde. Antibodies against pSTAT1 (pS727, 250 μg/ml), pSTAT2 (pY690, 500 μg/ml), pSTAT3 (pY705, 250 μg/ml), pSTAT4 (pY693, 250 μg/ml), pSTAT5a (pY694, 250 μg/ml) and pSTAT5b (pY699, 250 μg/ml) were purchased from BD Pharmingen (San Jose, CA). STAT3 inhibitor V (S3I) was obtained from CalBiochem/EMD biosciences (Gibbstown, NJ). Fluorescence was evaluated with a FACSCalibur flow cytometry and data was analyzed using FlowJo software (TreeStar, Ashland, OR).

### Psychometric Tests, Child-Pugh Scores, and MELD Scores

#### Psychometric Tests

MHE was diagnosed by the PHES battery, which has been regarded as the gold standard of MHE diagnosis^6^. PHES comprises a number of cognitive tests, including the Number Connection Test A and B, a Digit Symbol Test, a Serial Dotting Test and a Line Tracing Test. After testing, PHES scores were calculated and the patients were diagnosed as having MHE if their score was less than 4.

#### Child-Pugh Score

This test was used to assess the prognosis of chronic liver diseases, especially cirrhosis. There are five clinical measures involved, which are total bilirubin, serum albumin, prothrombin time, ascites and hepatic encephalopathy. Chronic liver disease prognosis could be classified into Child-Pugh classes A to C.

#### MELD Score

This is a scoring system for assessing the severity of chronic liver disorders, and includes measures such as serum bilirubin, creatinine, and clinical etiology.

### Statistical analysis

Averages of numerical variables were presented as 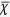 ± SD. Differences were compared by chi-square test for categorical data, and unpaired Student’s t-test for continuous roughly normally distributed data. Cytokine concentrations were compared by Mann-Whitney U test. Correlation between cytokine levels and seizure frequency and severity was analyzed by Spearman correlation and multivariate linear regression analysis (MLRA). In the MLRA, all continuous data had a skewed distribution and were therefore logarithmically transformed to fit a normal distribution. MRLA was done on the patient population with and without MHE. A best model discrimination was based on principle of squared minimums and greatest *r*^2^. All analyses were performed by SPSS software (version 12.0). A P value of <0.05 was considered to be statistically significant.

## Results

### Patient characteristics

The demographic and clinical data of 121 HCC patients and 60 healthy controls are collected and summarized in Table 1. There were 121 (66.9%) male and 60 (33.1%) female patients. The average duration of hospital stay for all involved patients was 17.9 ± 13.8 days. The HCC patients involved in this study were categorized into three groups; No MHE (n = 46), MHE (n = 48), and CHE (n = 27). Child-Pugh and MELD scores were calculated on each patient and are listed in Table 1. Laboratory findings are shown in Table 2.

### Correlation between cytokine levels and different stages of hepatic encephalopathy

To identify potential serum biomarkers to differentiate MHE, we examined the concentration of 14 serum cytokines: IL-1β, IL-1Ra, IL-2, IL-4, IL-6, IL-8, IL-10, IFNγ, IL-17a, IL-23, IFNλ1, IFNλ2, IFNλ3 and IFNλ4. The results of statistical analysis on each cytokine and ammonia are shown in Table 3. Hyperammonemia and significant inflammation have been previously reported to contribute to the development of hepatic encephalopathy, so ammonia concentrations were included in this analysis. Concentrations of all cytokines and ammonia were correlated with HCC and CHE. We found that only levels of IL-1β, IL-6, IFNγ, IL-17a, IFNλ2 and IFNλ3 were correlated with MHE (P = 0.014, <0.001, <0.001, <0.001, 0.034 and <0.001, respectively). To further evaluate the correlation of cytokines with MHE, all cytokines with a P < 0.1 (IL-1β, IL-6, IFNγ, IL-17a, IFNλ2 and IFNλ3) were included in an initial MLRA using MHE as dependent variable. IL-1β, IL-6, IFNγ, IL-17a and IFNλ3 were identified as significantly changed in MLRA, with P < 0.01 (P = 0.009, 0.007, 0.006, 0.008, 0.007, respectively, Table 4).

**Figure 1:**
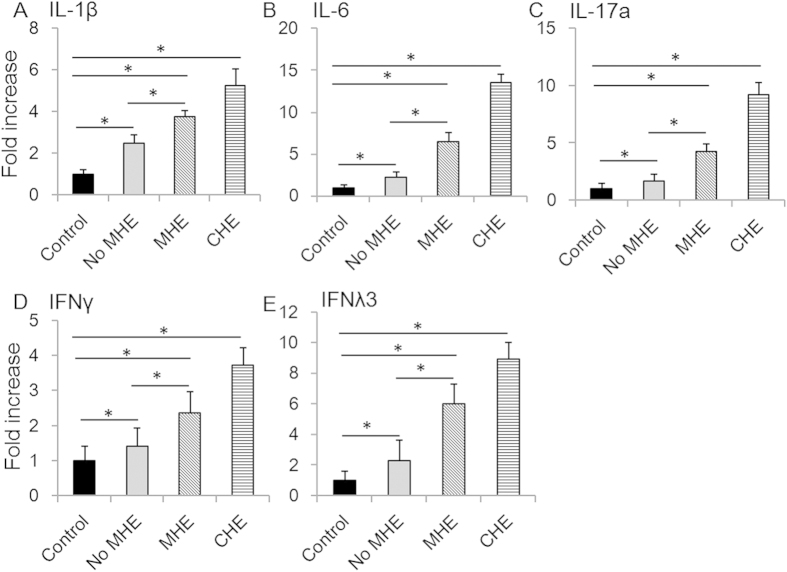
Protein levels of IL-1β, IL-6, IL-17a, IFNγ and IFNλ3 were elevated in serum of patients with MHE. The fold increases of protein concentrations of IL-1β (**A**), IL-6 (**B**), IL-17a (**C**), IFNγ (**D**) and IFNλ3 (**E**) were determined in all patients. Asterisks denote p<0.05 in statistics. MHE: minimal hepatic encephalopathy; CHE: clinical hepatic encephalopathy.

IL-1β and IL-6 levels were dramatically elevated in HCC patients (Fig. 1A,B, Table 3). IL-6 concentration was significantly higher in MHE patients than in patients without MHE. IL-6 levels were also higher in HCC patients without MHE than in controls. In addition, IL-6 levels were higher in patients with CHE than in patients with MHE (Fig. 1B, Table 3). Similar patterns were observed for IL-17a, IFNλ3 and IFNγ (Fig. 1C,D, Table 3). Levels of all IFNλs were elevated in HCC patients. Compared to IFNλ1, IFNλ2 and IFNλ4, IFNλ3 levels were much higher in all groups of patients.

Since circulating IL-6 and other cytokines tend to elevate when liver function deteriorates, we analyzed whether the relationship between IL-6 concentration and MHE was independent of liver function (MELD scores). As shown in Table 5, an MLRA using MHE as dependent variable and MELD, IL-1β, IL-6, IFNλ3, IFNγ and IL-17a as independent variables showed that IL-6, IL-17a and IFNλ3 had a predictive value (independent of MELD) for MHE (Table 5). This was not the case for IL-1β (P = 0.283) or IFNγ (P = 0.178). The above data indicate that IL-6, IL-17a, and IFNλ3 have predictive value for the diagnosis of MHE in patients with HCC.

**Figure 2:**
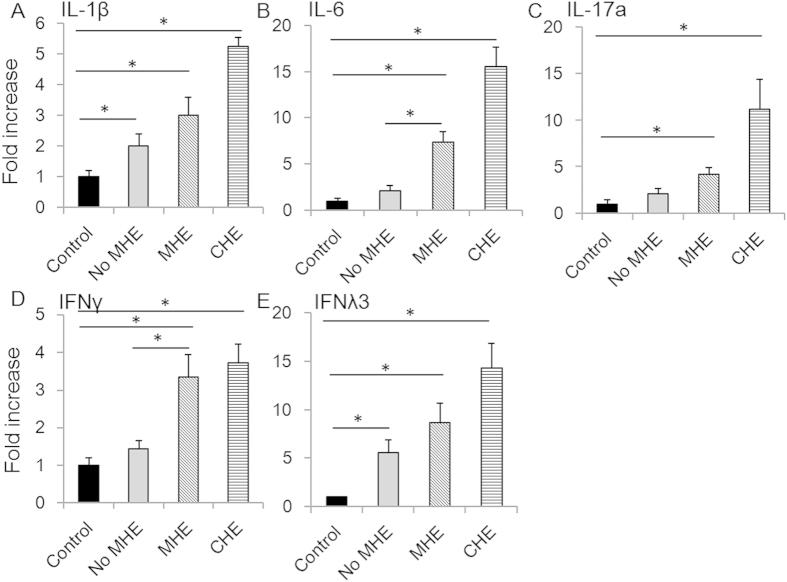
mRNA levels of IL-1β, IL-6, IL-17a, IFNγ and IFNλ3 were elevated in serum of patients with MHE. The fold increases of mRNA concentrations of IL-1β (**A**), IL-6 (**B**), IL-17a (**C**), IFNγ (**D**) and IFNλ3 (**E**) were determined in all patients. Asterisks denote p<0.05 in statistics. MHE: minimal hepatic encephalopathy; CHE: clinical hepatic encephalopathy.

The mRNA levels of all 14 cytokines were also evaluated, and the results for IL-1β, IL-6, IFNλ3, IFNγ and IL-17a are shown in Fig. 2. We found that, in contrast to protein levels, levels of IL-17a and IFNλ3 mRNA were not associated with MHE (P = 0.211 and 0.530, respectively). However, levels of IL-6 and IFNγ mRNA were associated with MHE (P = 0.019 and 0.030, respectively, Fig. 2). The mRNA levels of all other cytokines were not correlated with MHE (P > 0.05). The differences between protein and mRNA levels suggest that regulation of translational mechanisms may also play a role in the development of MHE.

### Correlation between STAT levels and MHE

**Figure 3:**
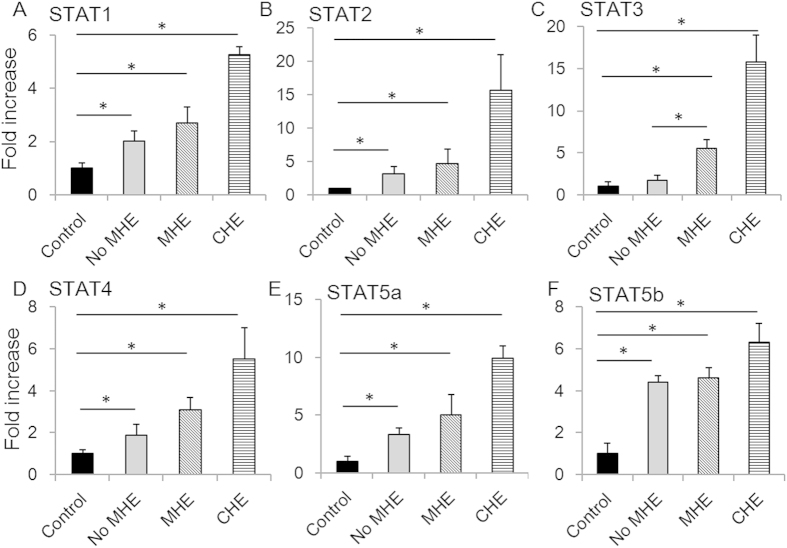
STATs were activated in MHE and CHE. The fold increases of concentrations of activated STAT1 (**A**), STAT2 (**B**), STAT3 (**C**), STAT4 (**D**), STAT5a (**E**) and STAT5b (**F**) were determined in all patients. Asterisks denote p < 0.05 in statistics. MHE: minimal hepatic encephalopathy; CHE: clinical hepatic encephalopathy.

Since IL-1β, IL-6, IFNλ3, IFNγ and IL-17a all activated the JAK-STAT pathway, we next investigated activation STAT protein family members in circulating PBMCs of patients at different stages of HE. All six STATs were strongly activated in CHE, compared to controls (P = 0.010, 0.013, 0.012, 0.021, 0.014 and 0.009, respectively, Fig. 3). In contrast, in MHE patients only STAT3 levels were associated with MHE in univariate and multivariate analysis when compared to patients without MHE (P = 0.005 and 0.008, respectively, Fig. 3). These results suggest that key cytokines conduct their signals via STAT3 in the development of MHE.

**Figure 4:**
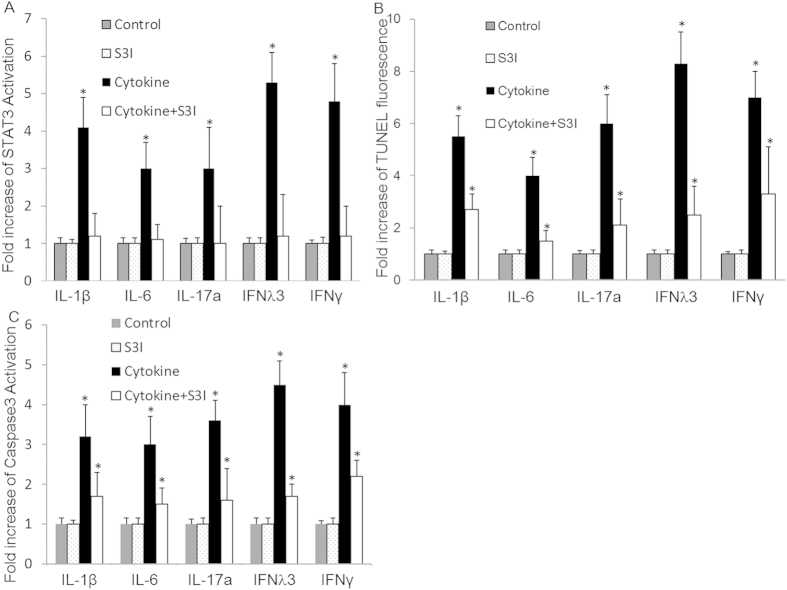
Cytokines-induced neural apoptosis in HCC is STAT3-dependent. Human neuron cells were left untreated (control) or treated with S3I alone or in combination different cytokine (IL-1β, IL-6, IL-17a, IFNγ or IFNλ3), respectively for 72 hrs. The levels of activated STAT3 were measured by flow cytometry (**A**). The levels of induced apoptosis were measured by TUNEL assay (**B**) and Caspase3 activation (**C**). Data represent a minimum of three independent experiments. Asterisks denote p < 0.05 in comparison to untreated samples.

Neural apoptosis in MHC was observed in a hepatic encephalopathy mouse model^24^. To investigate the role of STAT3 in the pathogenesis of MHE in HCC, we investigated activation of STAT3 and the effectiveness of a STAT3 inhibitor (S3I) by flow cytometry (Fig. 4A) and on apoptosis as measured by TUNEL assay in the presence and absence of STAT3 inhibitor in human neuron cells stimulated with IL-1β (200 ng/ml), IL-6 (10 ng/ml), IFNλ3 (100 ng/ml), IFNγ (100 ng/ml) and IL-17a (1 ng/ml). We found that the STAT3 inhibitor partially protected neuron cells from induced apoptosis (Fig. 4B,C). This data indicates that STAT3 contributes to neural apoptosis induced by cytokines in MHE.

Combinations of two cytokines from the group including IL-1β, IL-6, IFNλ3, IFNγ and IL-17a were used to treat neural cells with or without the STAT3 inhibitor, and apoptosis were detected by TUNEL assay. Our results indicated that all combinations exacerbated apoptosis, with the combination of IFNλ3 + IFNγ being the most effective, with an elevation of 50%. The STAT3 inhibitor effectively inhibited apoptosis induced by combinations of cytokines by at least 50%.

## Discussion

Circulating cytokines are key mediators of both inflammatory processes and immune surveillance against tumors. Previous studies have implicated various cytokines in HE and MHE^11^,^12^,^13^,^17^,^20^. In this study, we investigated the association of serum concentrations of 14 cytokines with MHE in HCC patients. Our data demonstrated seven potential risk factors for MHE in patients with HCC, including protein levels of IL-1β, IL-6, IFNγ, IFNλ3 and IL-17a, and mRNA levels of IL-6 and IFNγ. Our results also indicate that key cytokines in the development of MHE may conduct their signals through STAT3 signaling.

Previous studies have demonstrated the correlation between concentrations of certain cytokines and MHE. IL-6 is a pleiotropic cytokine expressed on various cell types and tissues^25^. Its serum concentration is elevated in the setting of many neurological disorders such as Alzheimer’s disease, trauma and meningitis^26^,^27^,^28^. Previous studies have shown elevated IL-6 levels in patients with HE or MHE^11^,^17^,^20^. In our study, serum IL-6 concentrations were associated with MHE and with CHE in HCC patients. In multivariate analysis, IL-6 concentration was positively linked to MHE, indicating that serum IL-6 may be a good candidate to indicate the presence of MHE in HCC patients (Fig. 1B and Tables 3, 4, 5).

It has been previously reported that IL-17a is involved in the development of many inflammatory diseases, and can be correlated with disease severity and outcome^29^. Another previous study indicated that interictal serum and CSF IL-17a levels were independent risk factors for seizure severity^30^. IL-17a is known to facilitate migration of active T cells, including Th17, across the blood-brain barrier, which aggravates seizure^30^. IL-17a has also been shown to play a key role in the pathogenesis of atherosclerosis and other vascular disorders^31^. Simon *et al.* have revealed a link between levels of circulating IL-17a and an increased risk of cardiovascular events^32^,^33^. These may all contribute to blood IL-17a elevation in our study (Fig. 1C and Tables 3, 4, 5). We believe that this is the first report of the correlation between IL-17a and MHE in HCC patients.

Both IL-17a and IL-6 have been correlated with severe liver conditions, such as liver fibrosis, which may contribute to MHE^34^. The subjects involved in this study all had HCC. It is expected that their IL-17a and IL-6 levels were changed significantly compared with those of general population. Although proper control groups were utilized in present study, there is still a possibility that our conclusions may be affected by this variability. Additional studies are needed to confirm our conclusions.

IFNλs are among the most recently described cytokines, and have been reported to be important for the pathogenesis, treatment response and outcome of viral and non-viral hepatitis^35^,^36^. Our findings in MHE were consistent with data from a previous study which described an elevation of IFNλ3 in CSF from patients with tick-borne encephalitis^37^. Studies of IFNλs on HE are limited. We analyzed levels of all four known IFNλs in HE and found a correlation between levels of IFNλ2 and IFNλ3 and MHE. We hope that our findings will benefit the clinical applications of this new group of cytokines. Further investigations are needed to confirm our conclusions.

There are limitations to this study. For example, all patients involved in this study were inpatients. Therefore, the condition of the patients tended to be more severe, and mild outpatient subjects were missed. This could introduce a bias to our results. Secondly, our study only identified key cytokines in HCC patients, and did not reveal any potential mechanisms. Further investigations are required to determine the mechanism by which these cytokines are correlated with MHE in HCC patients. In addition, whether neuronal apoptosis induced by elevated cytokines is a part of the pathogenesis of MHE, rather than a consequence of MHE, was not actually answered in this study. Additional research projects are needed to solve this issue.

In conclusion, among the 14 cytokines investigated, protein expressions of IL-1β, IL-6, IFNγ, IFNλ3, and IL-17a, and mRNA levels of IL-6 and IFNγ, were correlated with MHE in HCC patients. Three independent risk factors for MHE in HCC were identified, including serum protein levels of IL-6, IL-17a, and IFNλ3. These cytokines may act in the pathogenesis of MHE through the STAT3 signaling pathway. Importantly, levels of these cytokines in suspected cases of MHE could be used as potential markers to identify MHE in HCC patients^38^.

## Additional Information

**How to cite this article**: Wu, H. *et al.* Cytokine levels contribute to the pathogenesis of minimal hepatic encephalopathy in patients with hepatocellular carcinoma via STAT3 activation. *Sci. Rep.* **6**, 18528; doi: 10.1038/srep18528 (2016).

## Figures and Tables

**Table 1:** Clinical characteristic of HCC patients (n = 121).

n	Control	No MHE	MHE	CHE
	60	46	48	27
Male/Female	39/21	31/15	34/14	17/10
Age (mean±SD years)	42.1 ± 17.6	55.5 ± 16.6	56.5 ± 16.3	65.9 ± 15.8
Ascites	N/A	6	21	20
Child-Pugh				
A	N/A	33	17	0
B	N/A	9	20	16
C	N/A	4	11	11
MELD	N/A	8 ± 4.2	11 ± 4.1	18 ± 3.6
PHES	−0.6 ± 3.4	−1.7 ± 2.2	−5.6 ± 3.5	−8.9 ± 5.8

HE: hepatic encephalopathy, MHE: minimal hepatic encephalopathy, CHE: clinical hepatic encephalopathy; PHES: psychometric hepatic encephalopathy score.

**Table 2:** Laboratory findings.

	Control (n = 60)	No MHE (n = 46)	MHE (n = 48)	CHE (n = 27)
GLU (mmol/L)	7.0 ± 3.6	6.3 ± 3.5	7.1 ± 5.5	7.8 ± 6.9
WBC (10^9^/L)	8.3 ± 4.1	9.2 ± 3.7	9.1 ± 4.2	8.8 ± 3.8
Neutrophils (%)	59.5 ± 20.2	58.5 ± 21.6	62.2 ± 28.1	64.2 ± 22.6
Lymphocytes (%)	38.1 ± 15.5	37.2 ± 22.8	36.5 ± 19.8	39.9 ± 24.2
HB (g/dL)	120.1 ± 29.2	129.1 ± 33.2	117.4 ± 34.6	107.7 ± 25.7
PLT (10/L)	310.2 ± 113.5	288.1 ± 112.0	299.2 ± 161.1	273.8 ± 133.2
ALT (IU/L)	33.2 ± 6.5	65.1 ± 34.3	79.8 ± 53.7	123.3 ± 66.2
AST (IU/L)	26.7 ± 7.4	74.8 ± 48.7	87.4 ± 58.6	132.6 ± 71.7
CK-MB (IU/L)	19.2 ± 24.1	22.5 ± 29.4	27.1 ± 30.7	24.2 ± 23.6
CRP (mg/L)	4.3 ± 3.8	3.8 ± 3.5	4.6 ± 4.1	5.7 ± 4.6
LDH (U/L)	218.2 ± 75.1	241.4 ± 81.2	234.1 ± 65.8	242.5 ± 75.5
K (mmol/L)	4.1 ± 1.9	4.5 ± 1.4	4.7 ± 1.3	4.9 ± 1.7
Na (mmol/L)	130.6 ± 3.62	141.3 ± 5.5	133.3 ± 5.3	135.9 ± 7.6
CL (mmol/L)	100.5 ± 30.7	106.3 ± 45.2	115.9 ± 37.7	116.9 ± 41.6
Prothrombin time (s)	12.3 ± 3.0	18.8 ± 5.8	19.4 ± 6.3	19.1 ± 7.0
Bilirubin (mg/dL)	0.8 ± 0.6	2.8 ± 1.2	2.3 ± 0.8	2.7 ± 1.6
Ammonia (uM)	66.5 ± 34.3	161.2 ± 68.4	175.3 ± 54.6	178.8 ± 71.2

MHE: minimal hepatic encephalopathy, CHE: clinical hepatic encephalopathy, GLU: blood glucose, LYM: percentage of lymphocytes, ALT: alanine aminotransferase, CL: blood chlorine, WBC: white blood cell counts, CK: creatine kinase, CK-MB: creatine kinase-MB, CRP: C-reactive protein, LDH: Lactate dehydrogenase

**Table 3:** P Values of different parameters in different groups.

	No MHE *P* vs.Control	MHE *P* vs.Control	MHE *P* vs.No MHE	CHE vs.Control	Global ANOVA*P* value
IL-1β (pg/mL)	0.005	0.012	0.014	<0.001	0.019
IL-1Ra (pg/mL)	0.014	0.033	0.143	<0.001	0.005
IL-2 (pg/mL)	0.009	<0.001	0.675	<0.001	<0.001
IL-4 (pg/mL)	0.005	0.006	0.554	0.006	0.003
IL-6 (pg/mL)	<0.001	<0.001	<0.001	<0.001	<0.001
IL-8 (pg/mL)	0.012	0.018	0.116	<0.001	0.001
IL-10 (pg/mL)	0.011	0.025	0.952	0.005	0.016
IFNγ (pg/mL)	<0.001	<0.001	<0.001	<0.001	<0.001
IL-17a (pg/mL)	<0.001	<0.001	<0.001	<0.001	<0.001
IL-23 (pg/mL)	0.005	0.001	0.216	<0.001	0.008
IFNλ1 (pg/mL)	0.011	<0.001	0.199	<0.001	0.003
IFNλ2 (pg/mL)	0.002	0.005	0.034	<0.001	<0.001
IFNλ3 (pg/mL)	<0.001	<0.001	<0.001	<0.001	<0.001
IFNλ4 (pg/mL)	0.006	0.001	0.148	<0.001	0.002
Ammonia (uM)	0.023	0.002	0.231	<0.001	0.009

MHE: minimal hepatic encephalopathy, CHE: clinical hepatic encephalopathy.

**Table 4:** MLRA shows that serum IL-1β, IL-6, IFNγ, IL-17a and IFNλ3 levels have predictive value for the presence of MHE.

Variable	β	Standard Error	*P*
IL-1β	−0.293	0.372	0.009
IL-6	−0.454	0.232	0.007
IFNγ	−0.388	0.248	0.006
IL-17a	−0.566	0.342	0.008
IFNλ2	−0.432	0.784	0.053
IFNλ3	−0.342	0.211	0.007

Multiple linear regression: predictive value of IL-6, IL-17a and IFNλ3 on MHE in all patients.

**Table 5:** MLRA shows that serum IL-6, IL-17a and IFNλ3 levels have predictive value for the presence of MHE (MELD was included as an independent variable).

Variable	β	Standard Error	*P*
IL-6	−0.467	0.415	0.009
IL-17a	−0.643	0.102	0.007
IFNλ3	−0.598	0.223	0.007

Multiple linear regression: predictive value of IL-6, IL-17a and IFNλ3 on MHE in all patients. Dependent variable includes MHE. The regression analysis included IL-1β, IL-6, IL-17a, IFNγ, IFNλ3 and MELD as independent variables.
